# Direct financial assistance for improved maternal and child health data: a pilot study supporting the health management information system in Malawi

**DOI:** 10.1186/s12913-022-07680-5

**Published:** 2022-03-05

**Authors:** Mariame O. Ouedraogo, Madalitso Tolani, Janet Mambulasa, Katie McLaughlin, Diego G. Bassani, Britt McKinnon

**Affiliations:** 1 Centre for Global Child Health, The Hospital for Sick Children, 686 Bay Street, Toronto, ON M5G 0A4 Canada; 2 Dalla Lana School of Public Health, University of Toronto, 155 College Street, Toronto, ON M5T 3M7 Canada; 3 Amref Health Africa, Lilongwe, Malawi; 4 Kamuzu College of Nursing, University of Malawi, Blantyre, Malawi

**Keywords:** Health management information system, Maternal and child health, Pilot intervention, Malawi

## Abstract

**Background:**

The health management information system (HMIS) is an integral component of a strong health care system. Despite its importance for decision-making, the quality of HMIS data remains of concern in low- and middle-income countries. To address challenges with the quality of maternal and child health (MCH) data gathered within Malawi’s HMIS, we conducted a pilot study evaluating different support modalities to district-level HMIS offices. We hypothesized that providing regular, direct financial assistance to HMIS offices would enable staff to establish strategies and priorities based on local context, resulting in more accurate, timely, and complete MCH data.

**Methods:**

The pilot intervention was implemented in Mwanza district, while Chikwawa, Neno, and Ntchisi districts served as control sites given support received from other institutions. The intervention consisted of providing direct financial assistance to Mwanza’s HMIS office following the submission of detailed budgets and lists of planned activities. In the control districts, we performed interviews with the HMIS officers to track the HMIS-related activities. We evaluated the intervention by comparing data quality between the post- and pre-intervention periods in the intervention and control districts. Additionally, we conducted interviews with Mwanza’s HMIS office staff to determine the acceptability and appropriateness of the intervention.

**Results:**

Following the 10-month intervention period, we observed improvements in MCH data quality in Mwanza. The availability and completeness of MCH data collected in the registers increased by 22 and 18 percentage points, respectively. The consistency of MCH data between summary reports and electronic HMIS also improved. In contrast, 2/3 control districts noted minimal changes or reductions in data quality after 10 months. The qualitative interviews confirmed that, despite some challenges, the intervention was well received by the participating HMIS office. HMIS staff preferred our strategy to other conventional strategies that fail to give them the independence to make decisions.

**Conclusions:**

This pilot intervention demonstrated an alternative approach to support HMIS offices in their daily efforts to improve data quality. Given the Ministry of Health’s (MoH) interest in strengthening its HMIS, our intervention provides a strategy that the MoH and local and international partners could consider to rapidly improve HMIS data with minimal oversight.

## Background

Health management information systems (HMIS) are deployed to assist health offices in recording, storing, and processing health data [1]. These data guide public health programs and policies and the effective allocation and use of health resources [1, 2]. The information gathered within the HMIS also plays a critical role when planning health targets and assessing progress towards reaching national and international health targets. Researchers also use routine HMIS data to answer pertinent health system research questions [3].

Despite its critical importance, HMIS in most low- and middle-income countries (LMICs) remain inadequately used by policymakers, researchers, and non-governmental organizations (NGOs) [3, 4]. This has been partially attributed to concerns over the completeness, timeliness, and accuracy of data collected [5–8] In Malawi, according to Malawi’s National Health Information System Policy, the HMIS remains “fragmented and unable to generate quality information at the time they are needed” [9]. Commonly cited issues related to HMIS data quality include data duplication and loss, untimely submission of reports, data fabrication, incomplete data, and poor agreement between registers, monthly summary reports, and electronic reports [10–12].

Increased efforts to support and strengthen HMIS have been noted in LMICs, including Malawi. Common data quality improvement initiatives include training health workers and managers, identifying and training focal HMIS persons, organizing frequent HMIS data quality audits and review meetings, and increasing supportive supervision in low-performing health facilities [13–16]. Other major initiatives to facilitate the collection of high-quality HMIS data have focused on introducing electronic HMIS in health facilities and simplifying data reporting tools [16–18]. However, barriers to the success of past efforts to improve HMIS data quality have been observed [16]. Poor data quality has been shown to persist due to the high turnover of health staff, interruption of funding, and lack of resources [16]. In preparation for this study, we recognized, through visits to district-level HMIS offices and health facilities in four districts of Malawi, that the lack of material and financial resources to HMIS offices for training and supervision was the main limiter of successful execution of HMIS tasks. This challenge was apparent given the irregular and short-term involvement of the Ministry of Health (MoH) and NGOs supporting HMIS offices.

In response to the barriers to HMIS data quality noted in Malawi, we designed an intervention consisting of providing direct financial assistance to HMIS offices. This intervention was nested within a multi-country development project focused on improving maternal and child health (MCH) outcomes in Sub-Saharan Africa. It aimed to assess whether a direct financial assistance program implemented in district-level HMIS offices could result in improved MCH data quality in districts of Malawi. We hypothesized that this approach would give HMIS offices more autonomy and capacity to establish their strategies and set their priorities to obtain and maintain accurate, timely, and complete MCH reports from health facilities.

## Methods

### Aim of the study

We aimed to assess whether providing direct financial assistance to facilitate the operations of district-level HMIS offices in Malawi would lead to improvements in the quality of MCH data gathered within the HMIS, compared to other HMIS data quality improvement strategies carried out by external NGOs and the government.

### Study design

This pilot study used a non-randomized control group pretest/post-test design. It had three phases: a baseline (pre-intervention) data quality assessment, a 10-month implementation phase, and an endline (post-intervention) data quality assessment and qualitative evaluation.

### Study setting and participants

This study was nested within the Canada-Africa Initiative to Address Maternal, Newborn and Child Mortality (CAIA-MNCM), a four-year project aiming at improving maternal, newborn, and child health in Ethiopia, Kenya, Malawi, and Tanzania. This project was carried out by a consortium of organizations including Amref Health Africa, WaterAid, and Children Believe, with research, evaluation and capacity building support from the SickKids Centre for Global Child Health. In Malawi, the CAIA-MNCM project was implemented by Amref Health Africa in four districts: Ntchisi, in the Central Region; and Neno, Mwanza, and Chikwawa, in the Southern Region.

**Fig. 1:**
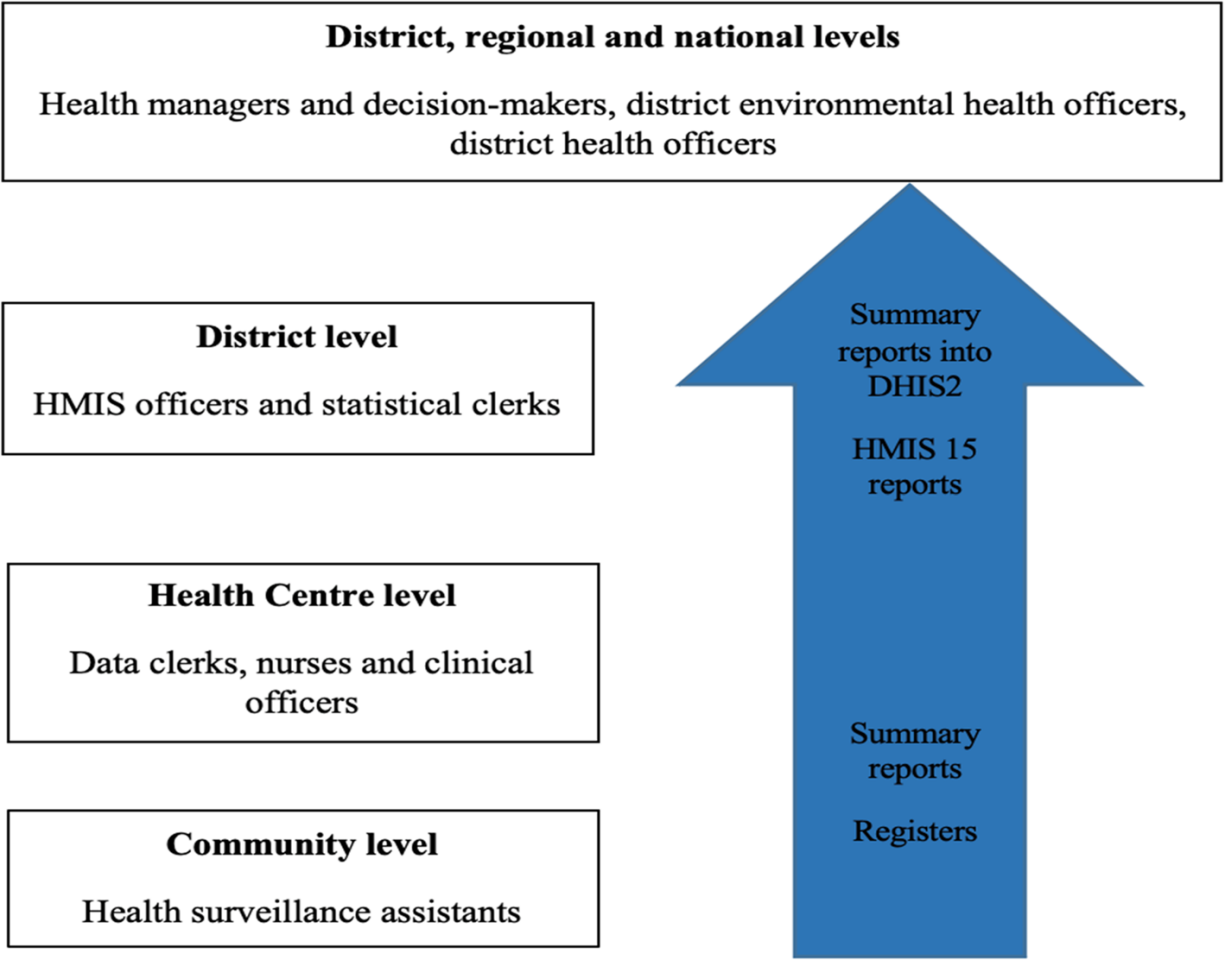
Flow of health information data in Malawi

Malawi uses the District Health Information System version 2 (DHIS2) software to manage its HMIS [19]. Reporting to Malawi’s DHIS2 is mostly paper-based in communities and primary health facilities. In communities, health surveillance assistants (HSAs) are responsible for recording all services provided into paper registers (Fig. 1). In health facilities, nurses, clinical officers, and data clerks maintain separate registers for various MCH-related various services (e.g., child health consultations, antenatal care, deliveries, etc.). On a monthly basis, data clerks combine data from their registers and the HSAs’ registers into summary reports and share those reports with their respective district-level HMIS offices. At that level, the reports are reviewed and entered into the DHIS2 system by the district statistical clerks and HMIS officers. Once in the DHIS2 system, the data are made available to decision-makers at district, regional, and national levels to inform health planning. District-level HMIS offices are also responsible for filling out HMIS15 reports, which extract specific policy-relevant indicators from all the summary reports sent by health facilities. The overall process at the HMIS offices is supervised by the HMIS officers and the district environmental health office (DEHO).

The study focused on the HMIS offices and the health facilities (i.e., health centers and district hospitals) that report data to these offices in the four districts of interest. Each district has one HMIS office and is served by at least one hospital and several smaller health centers. Neno has nine health centers, while 10, three, and 15 health centers are found in Ntchisi, Mwanza, and Chikwawa, respectively. Given the relatively large number of health facilities in Chikwawa, we randomly selected nine to participate in the study.

### Processes, intervention, and comparisons

The direct financial assistance strategy was implemented in Mwanza, while Chikwawa, Neno, and Ntchisi served as control sites. Mwanza was selected to pilot test this strategy because the HMIS office was not receiving external support from NGOs other than Amref Health Africa in Malawi. Additionally, during the study period, the HMIS office was receiving minimal support from the Mwanza district health office (DHO). The HMIS office in Mwanza consented to not receiving support from other NGOs during the study period to participate in the study. The HMIS offices in Neno and Chikwawa, on the other hand, were supported by different NGOs. Ntchisi served as a ‘pure’ control, given the absence of defined assistance from external partners to the HMIS office. However, Ntchisi did receive sporadic support from its DHO. In contrast, Neno and Chikwawa districts reported no support from the district-level MoH during the study period. Neno, Chikwawa, and Ntchisi represented suitable comparisons to assess the benefits and limitations of our intervention compared to more standard NGO strategies, which generally provide less autonomy to HMIS officers to choose and implement quality improvement activities, and to the absence of clear and consistent support.

#### Baseline data quality assessment

Before starting the intervention, we assessed the various MCH data in health centers, district hospitals, HMIS offices, and the DHIS2 platform. We reviewed registers and monthly paper and electronic reports to assess the quality of the information reported on antenatal care (ANC), maternity and postnatal care (PNC) services. We also reviewed HMIS15 reports, which capture a subset of indicators from the various summary reports.

We extracted the following MCH indicators from the different records: (1) first ANC visits, (2) facility-based deliveries, (3) breastfeeding within 60 min of birth, (4) postnatal care within 48 h (PNC48) offered to mothers, and (5) PNC48 for babies. We reviewed data collected between May and October 2018.

The quality of key MCH data within the HMIS was assessed considering four dimensions of data quality, as highlighted in the World Health Organization’s data quality review toolkit [20–22]:

1. Availability of relevant MCH registers and monthly reports: Number of registers/monthly reports available divided by the total number of registers/monthly reports expected to be available.
2. Timeliness of submission into DHIS2: Number of monthly reports entered into the DHIS2 on time (by the deadline for reporting) divided by the total number of monthly reports expected to be in the DHIS2.
3. Completeness of MCH registers and monthly reports: Number of MCH registers/monthly reports with non-missing values for selected MCH data elements divided by the total number of registers/monthly reports available.
4. MCH data consistency from health facilities to HMIS offices to the DHIS2 platform: a) percentage of health facilities for which the counts for selected MCH indicators in registers and the values of MCH indicators reported on the monthly reports found in the HMIS offices matched; b) percentage of health facilities for which the values of selected MCH indicators reported on monthly reports found in the HMIS offices and those found in the DHIS2 matched.

#### Data quality improvement strategy

The intervention consisted of transferring funds to Mwanza’s HMIS office following the submission of detailed monthly budgets and summaries of planned activities by the HMIS officers. Considering the amount that Mwanza’s HMIS office requests from its DHO management to maintain HMIS activities and what would be sustainable to the DHO, we established a fixed amount to allocate to the HMIS office for the duration of the study. The amount of 250,000 Malawian Kwacha (MWK) per month (equivalent to approximately 338 US Dollars as of December 1st, 2019) was provided for 10 months, between June 2019 and March 2020.

At the beginning of each month, HMIS officers developed and shared activity plans and budgets with the study investigators for review. The investigators verified that the activities were specific to HMIS strengthening and that the budget for the proposed activities did not exceed their monthly amount of 250,000 MWK. Once approved, the monthly allocation was transferred to a bank account opened specifically for cash withdrawals by the HMIS officers. At the end of each month, HMIS officers provided the study team with a summary of their activities, including a description of the location, duration, and targeted individuals or institutions. For further accountability, detailed costs, along with itemized receipts, were provided to the study team. The development of plans and reporting of activities did not result in additional work commitments for HMIS office staff, who are expected, under the right circumstances, to work collaboratively to plan and implement HMIS-specific activities and report to their DHO. The study team also conducted onsite monitoring visits to ensure activities were conducted. The HMIS office staff were blinded to the indicators being assessed in the study and could use the funds to implement activities to reach overall greater HMIS data quality.

In parallel to the data quality assessment, we conducted interviews on a quarterly basis with HMIS officers and representatives from implementing NGOs in the control districts. The discussions helped establish the activities in the comparison districts and highlight data quality strengths, challenges, and opportunities for improvement.

#### Endline evaluation

Following a 10-month intervention, trained research assistants visited health facilities and HMIS offices to repeat the data quality assessment. The post-intervention data quality assessment focused on data collected by the HMIS offices between August 2019 and January 2020. Additionally, we conducted in-depth interviews in Mwanza with relevant informants to assess their opinion on the district’s current performance in MCH data quality and their general perceptions of the intervention, specifically on the acceptability and appropriateness of the intervention [23]. Participants for the interviews were selected using purposive sampling, targeting HMIS office staff and primary data handlers from the district hospital and the three health centers.

### Data analysis

We had a limited number of data points (i.e., health facilities and hospitals) in each district: four in Mwanza, 10 in Neno, 11 in Ntchisi, and nine in Chikwawa. Given this, we estimated district-level median scores in availability, timeliness, completeness, and consistency of selected MCH indicators across different levels of data aggregation. Due to the limited sample size, our pilot study was not powered to assess the impact of our intervention on MCH data quality. Instead, we emphasize describing the changes in data quality observed between the baseline and endline assessments, keeping in mind differences that would be considered relevant.

Qualitative interviews were conducted in Mwanza either in English or Chichewa, depending on the participants’ preference. Participants were sampled purposively given their participation in the intervention and/or their role in HMIS data collection in the health facilities. Key respondents included the Mwanza’s HMIS officer (*N* = 1), the two district statistical clerks (*N* = 2), and the facility data clerks (*N* = 3). Qualitative data were collected using audio recording devices. Interviews conducted in Chichewa were translated into English at the point of transcription. Transcripts were coded using the NVivo software program to identify the main themes about the usefulness, perceived effectiveness, challenges, and sustainability of the intervention.

## Results

### Summary of activities

Over 10 months, Mwanza’s HMIS office carried out eight activities to enhance the availability, timeliness, completeness, and consistency of the data (Table 1). No implementation occurred in September and December 2019 due to delays in transferring funds from the study team office to the HMIS office’s bank account.

**Table 1:** Summary of activities conducted between June 2019 and March 2020 in the intervention (Mwanza district) and control sites (Chikwawa, Neno, Ntchisi districts) in Malawi

District	Activities
**Mwanza (Intervention site)**	• Purchasing of airtime and internet dongles to facilitate entry into the DHIS2 at the HMIS office • Printing and provision of data collection and reporting tools to health facilities • Data quality assessment of ANC, maternity, sexually transmitted infection (STI) and outpatient registers in health facilities • District- and facility-level orientations on new and revised data collection tools. • Supportive supervision in health facilities to review ANC, maternity, family planning, and HMIS15 data • District- and facility-level data reviews
**Chikwawa (Control site)**	• Allowances and transportation for HMIS office staff to perform supervision visits and data verification exercises in health facilities • Monthly airtime subscription of 10GB provided to the HMIS office to facilitate the submission of reports to DHIS2 • HMIS office staff provide access to the NGO office to print registers, summary sheets, and supervision checklists • Transportation of new logs from the National Statistical Office to Chikwawa • Deployment of data clerks in targeted health facilities
**Neno (Control site)**	• Monthly financial allocation for HMIS office to buy stationery • Creation of a booklet for tracking the submission of monthly reports by health facilities • Quarterly financial assignment to HMIS office to cover daily allowances and fuel costs for in-facility supervision visits • Internet bundles to facilitate timely data entry into DHIS2 • Deployment of data clerks in targeted health facilities
**Ntchisi (Control site)**	• Deployment of data clerks in targeted health facilities • Stationary (tonner, papers, writing materials) and airtime for data entry • Transport of monthly reports from facilities to HMIS office

One activity (purchasing airtime and internet dongles) specifically targeted the HMIS office to improve the timeliness of data entry into DHIS2, while the other activities aimed at reaching health facility-level staff. Through their various actions, the HMIS officers oriented the data clerks on data collection tools, assessed how they were entering the data into the registers and summary forms, and provided advice to improve reporting. Using the financial resources provided, the HMIS office supported MCH data, as well as data on outpatient services, infectious diseases, immunizations, palliative care, and youth-friendly services.

There were no major differences in the type of activities implemented in the intervention and control sites. While Ntchisi conducted their HMIS-related activities using sporadic support from the MoH, Chikwawa and Neno were supported by different international NGOs. In all districts except Ntchisi, there was a focus on improving the timeliness of data entry by securing an internet connection, and on conducting supervision visits to lower-level health facilities. Contrary to Mwanza, HMIS-related activities in the comparison districts did not include data quality assessment or reviews. While no important distinctions were noted in the type of activities conducted in the intervention and control districts supported by NGOs (Chikwawa and Neno), differences were observed in the frequency of activities. Using the funds provided by the study (i.e., 250,000 MWK per month (equivalent to approximately 338 US Dollars as of December 1st, 2019)), Mwanza’s HMIS office had to prioritize specific interventions, while the NGOs supporting Neno and Chikwawa determined the activities and financial allocations for the HMIS office during the study period. For example, a maximum of 300,000 MWK per month (equivalent to approximately 406 US Dollars as of December 1st, 2019) was allocated to Chikwawa’s HMIS office for allowances, transportation for supervision visits. For one round of similar activities across its health facilities, Mwanza’s HMIS office spent approximately 170,000 MWK (equivalent to approximately 230 US Dollars as of December 1st, 2019)). With this amount, the HMIS office allocated around 88,000 MWK on allowances (4000 MWK per participant) and the rest on supervisory sheets and fuel to move across their health facilities.

### Data quality assessment

**Fig. 2:**
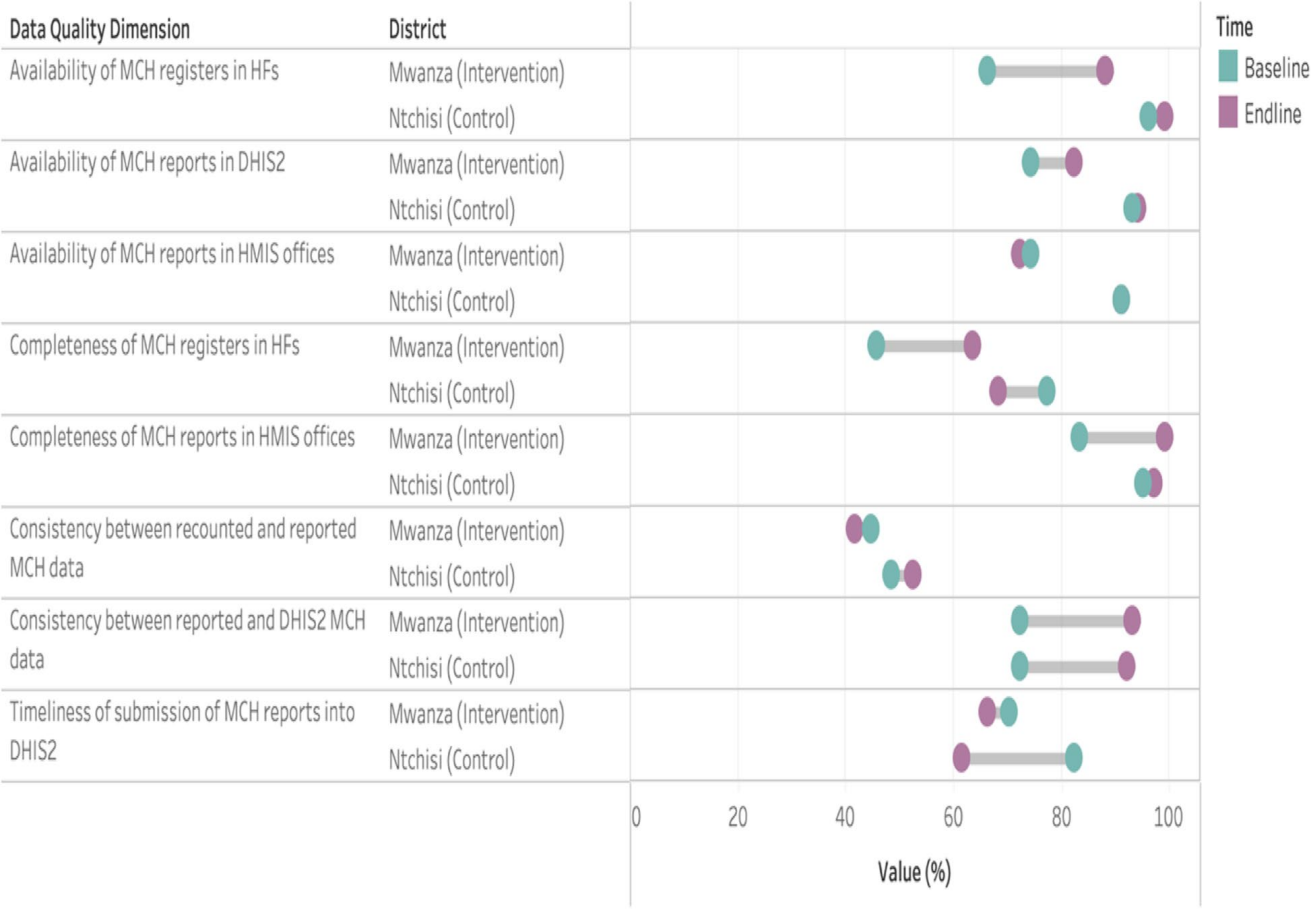
Changes in data quality dimensions between baseline (2018) and endline (2019/2020) in the intervention site (Mwanza) and the control district that received no support from NGOs (Ntchisi)

**Fig. 3:**
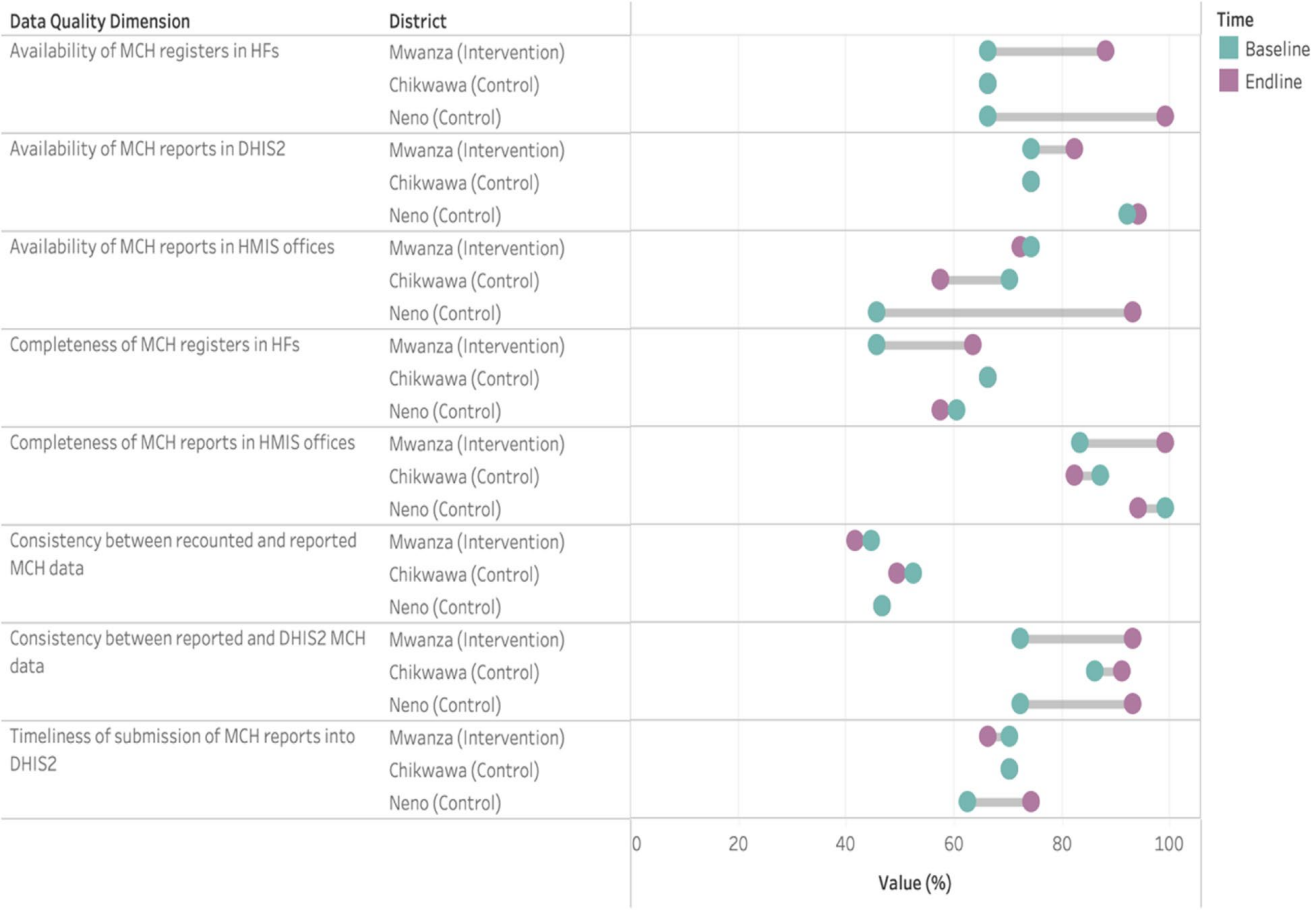
Changes in data quality dimensions between baseline (2018) and endline (2019/2020) in the intervention site (Mwanza) and the control district that received support from NGOs (Chikwawa and Neno)

Our findings highlighted changes in data quality following the 10-month intervention period in the intervention and control sites (Figs. 2 & 3). In Mwanza, improvements in the availability and completeness of MCH registers in health facilities increased by 22 percentage points (67% at baseline to 89% at endline) and 18 percentage points (46% at baseline to 64% at endline), respectively. The completeness of MCH reports sent to the HMIS office also improved from 84 to 100%. The availability of DHIS2 reports also increased from 75 to 83%. We noted no changes in the consistency of recounted and reported MCH data. In Mwanza, we also observed some progress in the consistency between monthly reports and DHIS2 entries (from 73% at baseline to 94% at endline). Compared to Mwanza, Ntchisi (the district that received no support from NGOs and relied on sporadic assistance from its DHO) had relatively high data quality indicators at baseline, specifically for the availability and completeness of monthly reports (Fig. 2). However, minimal improvements in data quality were observed for Ntchisi during the study period. This is in contrast to notable improvements in availability and completeness data quality indicators in the intervention district. We also noted a drop in the completeness of MCH registers and the timeliness of reporting into the DHIS2 in Ntchisi.

In Fig. 3, we show that Neno district, which has received continuous support from two NGOs (Partners in Health [24] and OPTIONS [25]) also reported important changes in data quality, with the availability of MCH registers increasing 33 percentage points and the availability of MCH reports at the HMIS office increasing 50 percentage points. In Chikwawa, where NGO support to HMIS office was less substantial than in Neno district, progress was only noted in the consistency between monthly report and DHIS2 data (87% at baseline to 92% at endline). Overall, in Chikwawa, minimal changes and, in some instances, reductions in data quality were noted.

### Qualitative assessment of the direct financial assistance strategy

#### Acceptability, appropriateness, and perceived effects on data quality

The respondents who participated in the intervention discerned the usefulness and potential of the direct financial assistance strategy to support HMIS data quality. As the funds dedicated to the HMIS office by the district health office were limited and no external partners provided additional support to the HMIS office, the study respondents appreciated the approach used in this study. In addition, the HMIS office staff reported that the project’s approach enabled them to do their job and gave them the flexibility to implement important HMIS-related activities.

*(The intervention) showed that we had a purpose, this has imparted us to do review meetings and (carry out activities) other than supervision.* **(HMIS officer)**

Through their activities, HMIS officers reported being better positioned to support statistical clerks in hospitals and health centers. Regular supervision visits and data quality assessments enabled them to assess whether data clerks were adequately entering the data into the registers and summary forms and provide feedback when needed. This is confirmed by the observed improvement in the completeness of MCH registers (Fig. 1) but contradicted by the findings related to the less consistent reporting from the registers to the monthly summary forms.

The HMIS office staff also highlighted that the direct financial assistance provided in the project enabled them to address gaps they had been observing in the district’s health facilities. For instance, in the past, data collection tools were supplied to the facilities without providing proper orientation to staff. The HMIS office staff reported now being able to spend time and resources to properly orient health workers, data clerks, and program coordinators on new and revised reporting tools. Given more frequent interactions with data clerks, the HMIS office staff highlighted being in a better position to recognize and address emerging data quality weaknesses.

*Orientation on data collection tools helped to give the people feedback on the gaps we had found and how they could fill them up. The review helped us to analyze the report that we had written after orientating them, and the data handlers could give their feedback on how we could best improve the report.* **(District Statistical Clerk)**

HMIS office staff believe that the intervention ultimately helped them improve their data quality through ensuring greater and more consistently available data collection tools and more frequent collaborations with facility data clerks. This is also reflected in the results of the DQAs, where we observed increased availability of reporting forms (registers and monthly summary forms) in health facilities. The support received by the HMIS office was also indicated during the interviews with the facility data clerks. However, although HMIS office personnel were reported to visit the health facilities, no improvements in the consistency between the data recounted from the registers and the data reported in the summary forms were noted.

*(The HMIS office people) come to supervise. They come to verify what we have sent them as original forms and if it is (agreeing) with the registers.* **(Facility data clerk)**

The approach used in this pilot study also revived HMIS office staff’s interest in their data and promoted better engagement and cooperation between the HMIS office and the various departments in hospitals and health centers. The HMIS office collaborated and communicated with the District Health Management Team (DHMT) through frequent data quality reports. The DHMT is a team composed of health workers and administrators responsible for setting health priorities at the district level.

*Because of this intervention, we had time to write the report after each activity and submit to the DHMT. Every month and quarter, we (could) come up with reports and submit to different people so that they know how they are doing in their program.* **(District Statistical clerk)**

Compared to previous support received to strengthen HMIS functions, the interviewees noted that our approach had the advantages of closely involving the individuals who are responsible for the data. They also highlighted the fact that other NGOs and the DHO could learn from this intervention, and the HMIS office’s ability to run activities with minimal funding.

*(The) intervention started from the grassroots, thus the data handlers, what they were facing as challenges [ … ]. I can say (this) intervention is better than the previous ones that just wanted to see the data but not concerned with the one collecting the data.* **(HMIS officer)**

*Not only the NGOs, but maybe even the DHO can borrow a leaf from what (this study) was doing to help us. That with the little we were getting from (this study), we were able to do some activities. [ … ] It’s the simple things; you can do a lot with 100,000 or 50,000 MWK.* **(HMIS officer)**

#### Challenges and unintended consequences

While respondents generally perceived improvements in HMIS data quality as a result of the direct financial assistance strategy, some challenges were also reported. There were unforeseen delays with the transfer of the monthly funds from the study team to the HMIS office, which resulted in delayed implementation. As a consequence of delayed transfer of funds from the study team end, no activities were conducted in September and December 2019.

*We could plan to do the activity in March, but we could be given the funds in April. [...] Like now, we have made a plan for February, but the money is not yet in.* **(HMIS officer)**

The amount of the monthly transfer was also determined as insufficient to carry out all activities needed to reach the desired level of data quality. Fluctuations in bank charges and fuel prices also significantly reduced the amount that the HMIS office was receiving every month. These unexpected charges prevented the HMIS office from realizing planned activities.

*We could write a proposal of about 250,000 MWK, and then we would add a bank charge of 30,000 MWK, you would find that the bank charge was more than what we thought, and we would get less than the amount we had planned for. As a result, we would cut some activities.* **(HMIS officer)**

These challenges ultimately prevented the HMIS office staff from proceeding with some activities and required them to be selective of which individuals, departments, facilities, and data collection tools to target.

*There are some programs that needed formal training and not just orientations. There are some data tools that were introduced, but people were just oriented and not trained. They just got a glimpse of the job, and that still compromises data documentation. Maybe if there can be a chance for them to be trained in different tools in data documentation.* **(HMIS officer)**

*[The study team] was not doing everything because they only provided a portion of the funding. Maybe when training coordinators, we were not training all of them, we were just taking the front-line workers who were the core coordinators.* **(HMIS officer)**

Echoing the concerns raised by the HMIS office staff, health facility-level data clerks also reported that some of the activities conducted by the HMIS office only reached a small portion of needed participants.

*The DQAs require everyone involved in the program to be present, but sometimes they just say we want the in- charge and other selected people. Everyone should be present, so they understand their program. Then maybe the programs can run smoothly.* **(Facility data clerk)**

The launch of the intervention had the unintended consequence of sparking tensions within the DHO and discordance over who would manage the funds. These concerns ultimately delayed the start of the intervention but were overcome following discussions between the investigators and the DHMT on the study and its intended benefits.

*When the (intervention) came, (some members of the DHMT) thought I would be very rich since they thought (the study team) would be providing a lot of money and even (some people were) not in agreement with me (participating in the study).* **(HMIS officer)**

#### Areas for improvement and sustainability

When asked about ways to improve our intervention, the respondents highlighted that a monthly allocation of 700,000 MWK instead of 250,000 would have been adequate (approximately 947 US Dollar instead of 338 (based on rate from December 1st, 2019)). Other recommendations included circumventing delays associated with the transfer of funds to the HMIS office’s bank account and settling the bank charges in advance of the study.

Sustainability by the district-level MoH is important to consider, particularly when support from NGOs is limited and unsteady. We therefore asked the interviewees if they thought this approach could be considered and adopted by the DHO. Money was identified as the main barrier that would prevent the DHO from considering this intervention in the long-term. Additional reasons challenging the sustainability of the project’s approach were the perceptions that the DHO may have on the value of the HMIS office in supporting and informing the health system.

*The limiting factor [...] could be money. We receive little money from the DHO. Sometimes they don’t (think about) HMIS because (they see it as) a preventive department. (The DHO) can’t give much money to HMIS, so they put it into other things. They are looking at assisting the sick people and not follow up on the data trends.* **(HMIS officer)**

*In the past, when HMIS came up with activities, they were removed by the DHO. They thought it was just a waste of resources forgetting that it is a good department where data can be accessed to help the hospital move forward.* **(HMIS officer)**

## Discussion

Improving and maintaining the quality of HMIS data is critical. Facilities’ failure to submit complete and accurate MCH reports to their district may result in a partial and incomplete representation of their MCH service provision. This could have important implications for the health of pregnant women, newborns, and children living in these districts, as the DHOs use information reported by health facilities to guide health planning and resource allocations. In this study, we explored an alternative strategy to support HMIS offices in their efforts to improve MCH data quality. To our knowledge, no study has attempted to evaluate improvements in the quality of HMIS data in response to direct financial assistance and autonomy provided to HMIS offices. Most of the initiatives conducted to improve HMIS data quality had defined activities such as introducing computer- and mobile-based HMIS and providing capacity-building activities for HMIS personnel [16]. Our pilot intervention aimed to give HMIS office personnel the flexibility to choose and implement activities to address HMIS data quality gaps in their health facilities. At the end of the 10-month intervention period, we noted progress in data quality in Mwanza. Our study shows that supporting HMIS offices with minimal yet regular funds can lead to improvements in health data quality. The study offers an alternative approach that NGOs could consider in order to strengthen HMIS data quality. Furthermore, by selecting a small monthly transfer, we aim to provide DHOs with evidence that HMIS functions can be maintained even when resources are limited. DHOs are supposed to allocate a certain portion of their budget to their HMIS offices, but they often do not due to resource constraints. This study provides initial support to the hypothesis that, in cases of limited budget, DHOs could still provide small monthly allocations so that HMIS offices can prioritize the implementation of certain activities.

In contrast, in Chikwawa, minimal changes in HMIS data quality were observed. The absence of improvements in data quality in Chikwawa may be explained by the fact that the HMIS office was limited in the number of facilities they could support using assistance provided by the NGOs. In fact, some hard-to-reach health facilities were entirely excluded from the HMIS support. An approach like the one piloted in this study could overcome such challenges by enabling HMIS office staff to target health facilities in critical need of support. Neno’s HMIS, on the other hand, was able to target all its facilities with support from their NGO partners. As a result, on many data quality indicators, Neno did better than Mwanza. Neno had the advantage of having two implementing partners, including one present since 2014 (Partners in Health). While there were no major differences in the types of interventions implemented in Neno and Mwanza, activities in Neno were implemented more frequently than in Mwanza, which likely explains why Neno overall had better MCH data quality compared to Mwanza. Furthermore, as highlighted during our quarterly interviews in Neno district, the HMIS office and their long-term NGO partner had established a strong model to carry out HMIS activities in their health facilities over the years. The progress noted in Neno suggests that an approach to assisting HMIS through long-term partnership and regular financial and technical support can also be very successful. However, the case of Neno is relatively rare, as long-term engagement in communities is not a reality for many NGO programs.

Although Ntchisi did not have any intervening partners, the overall quality of the data was high. Yet, some non-negligible reductions were noted for the completeness of registers and the timeliness of report entry into DHIS2. As identified by Ntchisi’s HMIS officer during the quarterly interviews, the absence of external partners and the limited assistance provided by the DHO hindered the HMIS office’s access to Internet services and their ability to secure transport and allowances for supervision visits. This indicates that regular support to HMIS offices is essential to prevent data quality loss.

Interestingly, in the intervention and control sites, we did not observe changes in the consistency between the MCH data recounted from the registers and reported in the monthly summary forms, highlighting the need for more targeted support in health centers. Our qualitative interviews in both the intervention and control districts provided indications that extracting some data from the registers, particularly the ANC and PNC registers, can be difficult for data clerks and medical professionals, given these registers follow women in cohorts (6 months for ANC and 3 months for PNC registers). The data handlers are required to determine in which cohort the women belong, based on their first ANC visit date. New and untrained professionals are more likely to assign ANC clients to the wrong cohort when aggregating the data. We also identified that, even when available, health facility personnel would sometimes refrain from using the PNC registers. The comparison districts also recorded low agreement between ANC registers and monthly reports, reinforcing the idea that facility-level health professionals and data clerks may face challenges when reporting ANC and PNC data on monthly summary forms and that more training and orientation opportunities should be provided.

Our quantitative results in Mwanza were also supported by our qualitative interviews. HMIS office personnel identified some advantages to our approach. Challenges were also noted, including the fact that the funds were deemed insufficient to run activities such as intensive training sessions in facilities, which could have helped improve the consistency of data from the registers to the summary reports. It is also possible that a longer intervention period, beyond 10 months could have led to better consistency of data reporting. In future studies considering this type of direct financial assistance strategy, efforts should be made to support the HMIS office for more than 10 months.

Although some improvements in MCH data quality were observed at the end of the intervention period, our pilot intervention had some limitations. Firstly, we implemented the intervention in a district with a small number of facilities, which might have eased the implementation of the strategy. The effects of our strategy may have been different in a district with a larger number of facilities with competing priorities for HMIS data quality. Secondly, due to the limited sample size, our pilot study was not powered to quantitatively assess the impact of our intervention on MCH data quality, compared with approaches implemented in the control districts. Thirdly, the qualitative interviews were of variable quality. Some of the responses were unclear, hence making transcription difficult. Although participants had the option of doing their interviews in Chichewa, some interviews were still done in English. This challenged some informants’ ability to answer the questions comprehensively. Lastly, as the endline data collection coincided with the first reported cases of COVID-19 in Mwanza, we missed the perspectives of additional key informants, including those from the DHO and the DEHO.

## Conclusions

This pilot intervention provides preliminary evidence that our approach – providing targeted funding directly to the HMIS office and letting staff determine and implement priorities for data quality improvement – can produce data quality changes in terms of the availability and completeness of data. Comparing data quality changes between the intervention districts and those that received fixed support from NGOs suggests that our intervention could lead to better, or similar improvements in data quality improvement than more standard approaches considered by NGOs to HMIS strengthening, at a considerably lower cost. Meaningful improvement in the consistency of reporting may require more time to allow HMIS officers to implement frequent and intensive activities such as training, supervision visits, and data quality assessments. Our approach empowered HMIS office personnel to set their own priorities to improve data quality. The strategy used in this study was preferred to more standard ways of supporting HMIS that provide less autonomy to HMIS officers to make decisions. Given the MoH’s focus on scaling up the performance of its DHIS2, NGOs and DHOs might consider implementing and evaluating this strategy of providing direct financial support with accountability measures in place at a larger scale in Malawi.

## Data Availability

The HMIS datasets used and analysed during the current study are not publicly available. The data belong to Malawi’s Ministry of Health and therefore cannot be shared by the authors due to legal and privacy restrictions. Also, the qualitative data generated and analysed during this study cannot be shared by the authors owing to the consent given by the participants, which limits data to the study team only.

## Abbreviations

ANC: Antenatal Care
CAIA-MNCM: Canada-Africa Initiative to Address Maternal, Newborn and Child Mortality
DEHO: District Environmental Health Office
DHIS2: District Health Information Software 2
DHMT: District Health Management Team
DHO: District Health Office
HMIS: Health Management Information System
HSA: Health Surveillance Assistant
LMIC: Low- and Middle-Income Country
MCH: Maternal and Child Health
MoH: Ministry of Health
NGO: Non-governmental organization
PNC: Postnatal Care
PNC48: Postnatal Care within 48 h
